# Dietary Guidance for Cardiovascular Health: Consensus and Controversies

**DOI:** 10.3390/nu15194295

**Published:** 2023-10-09

**Authors:** Panagiota Pietri

**Affiliations:** Athens Medical School, University of Athens, 11527 Athens, Greece; panpietri@hotmail.com

## 1. Introduction

Healthy diet, regular exercise and smoking cessation comprise the ‘golden triad’ of primary prevention of cardiovascular disease (CVD). These components are the fundamental lifestyle measures that may delay cardiovascular aging and promote longevity [1].

Many dietary models have been proposed to benefit cardiovascular and holistic health, with common as well as distinct constituents that differentiate them. Favoring fruits, vegetables, nuts, fish and legumes over red meat, refined carbohydrates and processed foods is the common substrate of the most popular diets. Among them, the Mediterranean diet, the DASH (Diet Approach to stop Hypertension) diet and other more or less identifiable diets are recommended as general guidance for health and longevity.

Although the dietary ‘portfolio’ is ample, comprising many approaches, most of which have been extensively studied, there is still a knowledge gap. Indeed, specific dietary guidance for sub-populations is missing (with some exceptions, such as the DASH diet for hypertensive patients. However, even in these populations, many dietary aspects are under scientific skepticism. Furthermore, the prognostic significance of micronutrient and vitamin supplementation is contentious.

Finally, data on the genetic, epigenetic and environmental factors that may modulate the relationship between diet and cardiovascular health are lacking and thus, these significant parameters have not incorporated into general dietary guidelines.

Future studies need to unravel the above interrelationships and upgrade the dietary guidance for cardiovascular health into optimal and personalized dietary approaches.

## 2. Dietary Models and Cardiovascular Health

Among several dietary patterns, the Mediterranean diet is the most thoroughly investigated and its prognostic value is unequivocal. Almost half a century ago, the landmark epidemiological study, ‘The Seven Countries Study’ by Ancel Keys et al., [2] demonstrated, for the first time, the beneficial effects of a diet prevalent in the Mediterranean on coronary artery disease and mortality. Indeed, the rural population of Crete, the island of Greece that was included in the study, had lower mortality rates from coronary artery disease compared to northern Europeans and North Americans—a finding that was attributed to the consumption of vegetables, legumes and olive oil, foods that defined the Cretan diet for many years [2]. Similar results were demonstrated for the Italian cohort of the study. A few years later, adherence to the Mediterranean diet was quantified with a score which was significantly associated with cardiovascular (CV) and total mortality [3]. Interestingly, the Ikaria study, an epidemiological study conducted in the Greek island of Ikaria, known for its long-lived population, showed a high adherence to the Mediterranean diet, which may in part be associated with the exceptional longevity of the inhabitants [1]. Apart from the known components of the Mediterranean diet [4], coffee consumption was also included in the diet of Ikaria’s centenarians [5].

Recently, a meta-analysis of 40 randomized trials with more than 35,000 participants demonstrated the superiority of the Mediterranean diet for the reduction of CV and total mortality [6]. Moreover, the Mediterranean diet was shown to be superior to low-fat diets for secondary prevention of CVD [7]. Apart from the established cardioprotective effects of the Mediterranean diet through several mechanisms such as the anti-oxidant and anti-inflammatory properties of its constituents, either alone or in combination, novel pathways of action have been proposed. Indeed, in a large-cohort study of women, closer adherence to the Mediterranean diet was associated with longer telomeres, a genetic marker of cardiovascular health and longevity [8]. Moreover, a recent study identified a metabolic signature that reflects adherence to the Mediterranean diet and predicts cardiovascular disease, independently of classic risk factors [9]. It is notable that the Mediterranean diet may also modify the relationship between CV risk and air pollution. Indeed, a prospective cohort study showed that individuals with close adherence to the Mediterranean diet had significantly lower rates of air-pollution-related CVD mortality [10].

According to a recent scientific statement from the American Heart Association (AHA), the Mediterranean diet, the DASH-style diet, pescetarian and ovo-lacto vegetarian diets align best with the AHA 2021 dietary guidance [11]. Very-low-fat diets and low-carbohydrate diets have low to moderate alignment, whereas ketogenic diets align poorly with AHA 2021 dietary guidance [11].

The DASH diet emphasizes the consumption of fruits, vegetables and low-fat diary products, similar to the Mediterranean diet, with greater focus on limiting sodium intake, given the beneficial effects on blood pressure and subsequent CV risk reduction [12]. Vegetarian diets, either excluding meat, poultry and seafood, or allowing diary products (lacto-) or eggs (ovo-), are associated with a reduced risk of CVD [13]. The ketogenic diet promotes the consumption of fat and the restriction of carbohydrates. Whether fats or carbohydrates exert the most detrimental effect on the vasculature and to whom is still under investigation. Previous and current European Society of Cardiology (ESC) guidelines for primary prevention of CVD encourage the replacement of saturated fats with unsaturated fats to reduce CV risk [14]. However, a recent review of the literature disputes the harmful effect of saturated fat on cardiovascular health, concluding that saturated fat *naturally* occurring in nutrient-dense foods can be safely included in the diet [15]. Recently, the PURE investigators suggested a diet composed of larger amounts of fruit, vegetables, nuts, legumes, fish and *whole-fat* dairy as a healthy diet associated with lower CVD and mortality, especially in countries with lower income [16].

The association of increased incidence of diabetes mellitus II and CVD with a high carbohydrate intake is well established. In a recent study, CV risk was found to increase after systematic consumption of foods with a high glycemic index [17]. The adverse effects of sugar-sweetened beverages in the epidemic of obesity and CVD has also been highlighted [18]. Sweetened beverages and refined carbohydrates are among the food categories with high pro-inflammatory potential, which may partly account for their harmful effects on cardiovascular health [19]. Furthermore, the dose–response relationship of carbohydrate intake and CV risk has been elucidated by a recent meta-analysis, which showed that the increased CV risk associated with carbohydrate intake was exacerbated when more than 60% of total energy was derived from carbohydrates, especially in Asian populations [20]. Current ESC guidelines for primary prevention recommend the restriction of free sugar consumption, particularly sugar-sweetened beverages, to a maximum of 10% of daily energy intake [14]. Hyperinsulinaimia is among the main mechanisms that may independently explain part of the increased CV risk associated with high glucose intake [21]. Atherogenic hyperlipidemia (defined as increased levels of triglycerides, low levels of HDL cholesterol and normal levels of LDL cholesterol, which is composed of small, dense particles), prevalent in obese and overweight patients with abnormal glucose metabolism, is also associated with increased CV risk and may explain part of the residual CV risk, beyond LDL cholesterol [22]. Interestingly, researchers have found that the remnant cholesterol, not LDL cholesterol, is associated with incident cardiovascular disease in patients at high cardiovascular risk, including diabetic patients [23,24].

**Figure 1:**
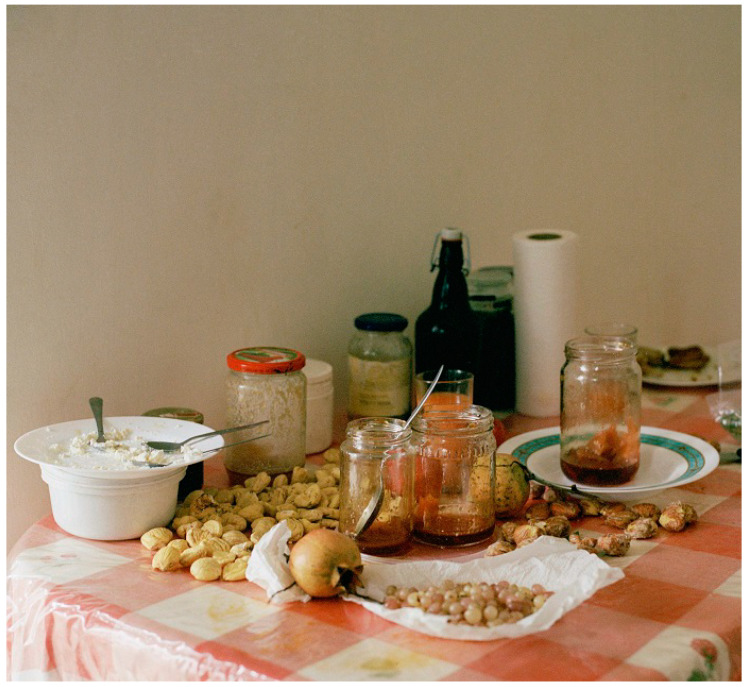
The mediterranean diet, as it is captured by the camera of the photographer Elena Heatherwick, in Crete, the largest island of Greece. Fruits, legumes, dairy products, honey and olive oil are included in the Cretan diet. *Taken after permission from a photography exhibition Foodprint, held in the National Museum of Contemporary Art, Athens, Greece, to be held in autumn 2023, as a tribute to the Mediterranean diet*.

To summarize, current evidence and international recommendations support the beneficial role of certain dietary patterns to promote cardiovascular and holistic health. The consensus remains in favor of the Mediterranean diet (Figure 1), both in American and European scientific statements and guidelines. Other similar diets are also encouraged. Red meat, processed food and refined carbohydrates should be restricted or avoided. Whether or not low- or whole-fat diary products should be incorporated is as yet contentious. In the future, the optimal dietary guidance for a healthy life, free of CVD and other chronic diseases, will be individualized, depending on baseline CV risk, genetic background, epigenetic modifications and environmental impact.

## 3. Micronutrients and Cardiovascular Health

Micronutrients, particularly minerals and vitamins, are essential for several cellular and molecular biological processes in the human body. Components such as sodium, potassium and magnesium are principal elements for normal myocardial function. Disturbance of the equilibrium between absorption, metabolism and excretion of these elements either inherently or in the course of a disease may have detrimental effects for cardiovascular health.

Excess sodium intake is associated with high blood pressure and subsequent increased CV risk [12]. Current European Society of Hypertension Guidelines recommend restriction of sodium intake to <2 g, corresponding to 5 g of salt, for patients with arterial hypertension [25]. Moreover, in adults with hypertension consuming a high sodium diet, salt substitutes replacing part of salt with chloride potassium are preferable [25]. The same level of salt restriction, lower than 5 g intake per day, is recommended for primary prevention of CVD, according to current ESC guidelines [14]. A large epidemiological study conducted in the general population from 21 countries, after 8 years of follow-up, demonstrated a positive association of major cardiovascular events with sodium intake only in communities where mean sodium intake was >5 g per day [26]. On the contrary, no association was observed for mean sodium intake of 4.7 g whereas an inverse association between CVD and salt intake was observed for mean sodium intake of 4 g/day, with authors to suggest that salt restriction policies may be more appropriate for the communities with the highest sodium intake than the others [26].

Interestingly, a recent analysis of the relationship of salt intake with life expectancy from 181 countries worldwide showed a positive correlation of sodium intake with life expectancy and an inverse association with all-cause mortality—an intriguing finding which implies that dietary sodium may not be as strong a culprit for adverse events as it was previously believed [27]. However, the researchers were skeptical to translate this information into clinical practice and dietary interventions.

Presumably, the recommended level of the optimal dietary salt intake should be revisited and guidelines distinct for general population and patient subpopulations should be released.

Magnesium, nature’s physiologic calcium blocker, is fundamental to myocardial function and cardiovascular health [28]. In the Framingham Offspring Study, a higher intake of magnesium and potassium, in conjunction with an unaltered sodium intake, was associated with reduced CV risk [29]. Moreover, in a Mendelian randomization analysis, genetically higher magnesium concentrations were found to be related to a reduced risk of cardioembolic stroke [30]. Magnesium has been implicated in the pathophysiology of several diseases, including arterial hypertension, diabetes mellitus and atrial fibrillation, while its deficit may accelerate cardiovascular aging through oxidative stress and inflammatory activation [31]. Although potassium intake is encouraged for blood pressure reduction, no recommendations exist for magnesium supplementation.

Vitamins, especially those with antioxidant properties such as vitamins C, E and D, are associated with cardiovascular health. However, the prognostic significance of vitamin supplementation is debatable. In the past, several prospective cohort studies investigating the effect of vitamin C and vitamin E supplementation on CV risk obtained conflicting results, ranging from an inverse association for vitamin C [32] to an inverse association for vitamin C and a positive association for vitamin E [33] or no association [34] between vitamin C supplementation and CV risk and mortality. Vitamin D, a vitamin with pleiotropic effects, is beneficial for cardiometabolic health. Indeed, vitamin D deficiency is associated with a high incidence of arterial hypertension, diabetes mellitus and lipid disorders, while it is related to poor cardiovascular prognosis [35]. Despite its significant prognostic value, the beneficial effects of vitamin D supplementation remain elusive. A large meta-analysis of 83,000 patients did not establish a significant association between vitamin D supplementation and reduced major cardiovascular events [36]. Methodology aspects regarding the assessment of vitamin D levels, such as the type of vitamin D that measured (25(OH)D or 1,25(OH)_2_D) or the method of measurement and seasonal variations, are challenging and may partly contribute to the conflicting results.

In accordance with other studies, a meta-analysis of 884 intervention trials evaluating 27 types of micronutrients showed divergent results with no effect on CVD for vitamin D, vitamin C and vitamin E supplementation, and modest- to high-quality evidence of beneficial effects of magnesium, coenzyme Q10, melatonin and *n*-3 fatty acid supplementation [37]. Considering coenzyme Q10, a randomized trial in patients with heart failure showed that Q1O supplementation reduced the risk of major cardiovascular events by 42% [38], while in elderly individuals, supplementation with 200 mg/day of Q10 combined selenium led to a significant decrease in blood levels of inflammatory and oxidative biomarkers [39]. Melatonin, a neuroendocrine hormone secreted by the pineal gland, is a principal regulator of the circadian rhythm [40]. Beyond this role, melatonin exerts anti-oxidant and anti-inflammatory effects which may benefit cardiovascular health [41].

In future, well designed, randomized, controlled studies are needed to investigate whether supplementation of Q10, melatonin or other ‘micronutrients’ may be included in the dietary guidance for cardiovascular health.

**In conclusion,** the optimal dietary guidance for cardiovascular health is still under investigation.

Although there is consensus for dietary patterns that have been proven to be effective in reducing cardiovascular risk, there is still a knowledge gap. This notion is reinforced by the fact that CVD remains the leading cause of death worldwide. Adherence to a healthy diet might be a problem, especially in low-income countries, but more influential and appropriately adjusted dietary interventions may be needed.

In the upcoming Special Issue entitled ‘Dietary guidance for cardiovascular health’, we will attempt to shed more light on this important field.

Authors are encouraged to submit their original research and review articles, contributing to the shaping of future direction.
